# The Bacterial Microbiome of the Tomato Fruit Is Highly Dependent on the Cultivation Approach and Correlates With Flavor Chemistry

**DOI:** 10.3389/fpls.2021.775722

**Published:** 2021-12-24

**Authors:** Carolina Escobar Rodríguez, Johannes Novak, Franziska Buchholz, Pia Uetz, Laura Bragagna, Marija Gumze, Livio Antonielli, Birgit Mitter

**Affiliations:** ^1^FFoQSI GmbH – Austrian Competence Centre for Feed and Food Quality, Safety and Innovation, Tulln, Austria; ^2^Institute of Applied Botany and Pharmacognosy (IAB), Veterinary University of Vienna, Vienna, Austria; ^3^Center for Health & Bioresources, Bioresources Unit, AIT Austrian Institute of Technology GmbH, Tulln, Austria

**Keywords:** tomato fruit, bacterial microbiota, organoleptic properties, aroma and flavor, hydroponics

## Abstract

The modes of interactions between plants and plant-associated microbiota are manifold, and secondary metabolites often play a central role in plant-microbe interactions. Abiotic and biotic (including both plant pathogens and endophytes) stress can affect the composition and concentration of secondary plant metabolites, and thus have an influence on chemical compounds that make up for the taste and aroma of fruit. While the role of microbiota in growth and health of plants is widely acknowledged, relatively little is known about the possible effect of microorganisms on the quality of fruit of plants they are colonizing. In this work, tomato (*Solanum lycopersicum* L.) plants of five different cultivars were grown in soil and in hydroponics to investigate the impact of the cultivation method on the flavor of fruit, and to assess whether variations in their chemical composition are attributable to shifts in bacterial microbiota. Ripe fruit were harvested and used for bacterial community analysis and for the analysis of tomato volatiles, sugars and acids, all contributing to flavor. Fruit grown in soil showed significantly higher sugar content, whereas tomatoes from plants under hydroponic conditions had significantly higher levels of organic acids. In contrast, aroma profiles of fruit were shaped by the tomato cultivars, rather than the cultivation method. In terms of bacterial communities, the cultivation method significantly defined the community composition in all cultivars, with the bacterial communities in hydroponic tomatoes being more variable that those in tomatoes grown in soil. Bacterial indicator species in soil-grown tomatoes correlated with higher concentrations of volatiles described to be perceived as “green” or “pungent.” A soil-grown specific reproducibly occurring ASV (amplicon sequence variants) classified as *Bacillus* detected solely in “Solarino” tomatoes, which were the sweetest among all cultivars, correlated with the amount of aroma-relevant volatiles as well as of fructose and glucose in the fruit. In contrast, indicator bacterial species in hydroponic-derived tomatoes correlated with aroma compounds with “sweet” and “floral” notes and showed negative correlations with glucose concentrations in fruit. Overall, our results point toward a microbiota-related accumulation of flavor and aroma compounds in tomato fruit, which is strongly dependent on the cultivation substrate and approach.

## Introduction

Tomato (*Solanum lycopersicum* L.) is the highest value fruit crop worldwide (Tieman et al., 2017) and major dietary source of valuable vitamins, minerals and antioxidants. Based on the increased global demand of fresh produce in the recent years, sustained efforts have been pursued in order to increase yield and fruit size, minimize pest susceptibility and therefore provide the consumer with year-round fresh produce. Such efforts include breeding programs which have extensively improved certain fruit qualities at the expense of aroma, taste, and nutritional value (Maul et al., 2000; Tieman et al., 2017; Wang and Seymour, 2017). For example, due to the deterioration of the commercial tomato flavor, which goes hand-in-hand with reduced consumer acceptance in the recent years (Klee, 2010), a growing trend in research emerged aiming at elucidating the causes underlying this phenomenon.

Flavor is described as a complex interaction between volatile and non-volatile compounds, which are perceived by retronasal olfaction and by the gustatory receptors on the tongue, respectively. These interactions and perceptions can be enhanced by orthonasal olfaction (“smelling”) as well as by visual and textural signals in the brain (Spence, 2015). Fresh tomatoes possess a characteristic sweet-sour flavor due to the presence of a complex mixture of volatile compounds that interact with sugars and acids in the fruit (Baldwin et al., 2008). Sugars are important compounds in regard to fruit yield, quality and organoleptic properties (Quinet et al., 2019), as they provide sweetness and are key in the generation of turgor pressure to promote cell expansion, ultimately affecting fruit size (Kanayama, 2017). In tomato fruit, sugars (primarily fructose and glucose) as well as acids (malic and citric acid) are the main contributors to taste (Tieman et al., 2012). Accordingly, high sugar and relatively high acid content are required for a favorable taste and several studies have shown that sweeter tomatoes are more acceptable (Malundo et al., 1995).

With respect to aroma, up to 400 organic volatiles have been identified in the tomato fruit, but only a fraction correlated with consumer liking and/or were associated with flavor intensity (Tieman et al., 2017). These volatiles derive from essential dietary nutrients like fatty acids, amino acids and colored carotenoids (Goff and Klee, 2006; Zanor et al., 2009). Furthermore, several studies demonstrated that the amount of some volatiles enhance the perception of sweetness in tomato fruit, increasing their likability (Baldwin et al., 2008; Tieman et al., 2012).

Genetic factors are responsible for the variability in the volatile profiles of tomato fruit. It was recently demonstrated that modern commercial cultivars show poor flavor compared to heirloom and wild accessions (Tieman et al., 2017). Intriguingly, despite the low rate of DNA sequence diversity among modern commercial tomato cultivars, Tieman et al. (2012) observed a variation in volatile content of as much as 3,000-fold across cultivars, possibly reflecting deviations in cultivation methods and post-harvest practices among lots (Tieman et al., 2012).

Hydroponic cultivation in greenhouses is considered effective in increasing productivity by unit land, as it allows extending the duration of the cropping season while minimizing the use of resources and negative environmental impacts (FAO, 2013). However, it is a widespread notion among consumers that tomatoes grown in hydroponics have bland flavor and aroma compared to those cultivated in soil (Padilla et al., 2007).

In the recent years, plant-associated microbial communities have taken a spotlight in research for their potential to impact plant health and, consequently, crop productivity and quality (Coleman-Derr and Tringe, 2014). Microorganisms are an integral part of the composition of fruits and vegetables and are found as epiphytes on the surfaces as well as endophytes within tissues (Droby and Wisniewski, 2018). Besides their roles in nutrient acquisition, stress alleviation as well as pest and disease control, the activities of plant microbiota can also influence the composition of secondary metabolites in their hosts (Berg et al., 2016), which include precursors of flavor-relevant compounds in fruit such as flavonoids and carotenoids (Robards and Antolovich, 1997; Kochevenko et al., 2012). This microbial-mediated modulation of fruit flavor has been demonstrated with methylotrophic bacteria in strawberry (Verginer et al., 2010) and with rhizobacteria in Basmati rice (Deshmukh et al., 2016). Furthermore, it has been proposed to be key in the taste of grapes and ultimately, the organoleptic characteristics of wine (Gilbert et al., 2014; Bokulich et al., 2016). Recently. changes in fruit quality and aroma have been linked to shifts in the fruit-associated bacterial community composition in raspberry (Sangiorgio et al., 2021). Compared to soil, hydroponic greenhouse cropping systems are an ecosystem with low microbiological complexity. Consequently, the microbial communities of tomato plants in hydroponics is less diverse than of those cultivated in soil (Rosberg et al., 2014). Therefore, along with the intrinsically impaired flavor of modern tomato cultivars, it is possible that the cultivation method could indirectly play a crucial role in the flavor of tomato fruit.

In this study, we intended to shed light on the impact of the cultivation method in the bacterial assemblages of ripe tomato fruit and how these communities correlate with tomato aroma and flavor profiles. With this aim, we cultivated five different commercial tomato cultivars (‘Ardiles,’ ‘Campari,’ ‘Cappricia,’ ‘Savantas’ and ‘Solarino’) in soil and in hydroponics. Ripe fruit were harvested and used for bacterial community analysis by 16S rRNA gene amplicon sequencing and HS-SPME GC/MS (Headspace Solid-Phase Micro-Extraction coupled with Gas Chromatography and a Mass Spectrometer) analytics for tomato aroma components, and GC-MS for flavor compounds. We described the various aroma, flavor as well as the bacterial community profiles for each cultivar-cultivation method combination and identified associations of microbial taxa and fruit organoleptic characteristics.

## Materials and Methods

### Cultivation and Harvesting of Tomato Fruit

**FIGURE 1:**
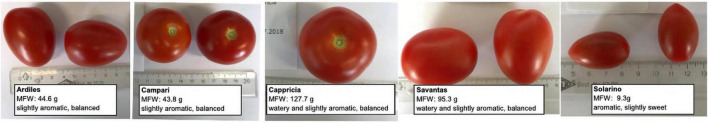
Fruit of five tomato cultivars ‘Ardiles,’ ‘Campari,’ ‘Cappricia,’ ‘Savantas’ and ‘Solarino.’ Shown are the mean fresh weight (MFW) and the scoring obtained from a trained tasting panel regarding the level of aroma perception and sugar/acid ratio. Scoring details are listed in Supplementary Table 1.

Seeds of five tomato (*Solanum lycopersicum* L.) cultivars with different organoleptic properties, namely ‘Ardiles,’ ‘Campari,’ ‘Cappricia,’ ‘Savantas’ and ‘Solarino’ were provided by the Viennese LGV Sonnengemüse cooperative (Figure 1 and Supplementary Table 1). To obtain the fruit material needed for this study, the tomato plants were cultivated under two different approaches: (1) in soil and (2) in hydroponics.

Cultivation in soil was started by sowing seeds in trays filled with potting substrate (Profi Substrat, Einheitserde special, Sinntal-Altengronau, Germany). After three weeks, seedlings were planted into 1.5 L pots filled with potting substrate, treated with organic-mineral NPK (5% + 4% + 8%) fertilizer (Gärtner Exclusiv Bio-Tomatendünger, *gpi green partners international* GmbH & Co. KG, Gladbeck, Germany) in a concentration of 2.2 kg/10 m^2^ and further kept in the greenhouse. After another three weeks, the tomato plants were transplanted into larger pots (12 L) filled with potting substrate placed in a wire-house, allowing for cultivation in semi-field conditions. Plants were watered whenever needed with tap water. Beginning nine weeks after sowing we treated each plant every two weeks with 1 L calcium nitrate greenhouse grade fertilizer (Haifa Cal CC) (Haifa Group, Israel) in a concentration of 33 g/L to prevent blossom end rot. Fourteen weeks after sowing, we fertilized the plants a second time with organic-mineral NPK (5% + 4% + 8%) solution.

For cultivation in hydroponics, seeds were sown on autoclaved rockwool plugs (2.5 cm × 2.5 cm × 4 cm) (Grodan, Roermond, Netherlands), which were pre-soaked in a diluted Complete Nutrient Solution for young plants (CNS, recipe in Supplementary Material) and placed into plastic Stervient High Containers (10.7 cm × 9.4 cm × 9.6 cm), with a plastic cover to prevent drying, and maintained at a temperature of 25°C in a Memmert Climatic Chamber until germination. After three days the containers were put into the greenhouse and rockwool plugs were watered daily with CNS. Two weeks after sowing, tomato plants in rockwool plugs were inserted into autoclaved rockwool cubes (10 cm × 10 cm × 6.5 cm) previously soaked in CNS and three cubes each with plants of the same tomato cultivar were placed on a tray to prevent exchange of microbiota between cultivars. Automated irrigation with a drip system was installed by inserting one drip into each rockwool cube and plants were drip irrigated with CNS for young plants. At this stage, plants had 3–4 leaves and were 15–20 cm high. Forty days after sowing, the rock wool cubes were placed on cocopeat mats (GBC-Österreich e. Gen. GartenBauCentrum, Wels, Austria), which were prior drip irrigated with nutrient solution for three days to achieve the necessary increase in volume (100 cm × 15 cm × 10 cm). From then on plants were irrigated with CNS for cocopeat mats.

Tomato fruit were harvested, when a single branch of a plant yielded at least 150 g of ripe fruit material. Fruit were considered ripe when they developed full red color. The fruit were then picked with gloved hands, put into sterile plastic bags and immediately further processed in the lab. Tomato fruit of five individual plants per tomato cultivar and cultivation condition were sampled. This resulted in 50 tomato samples [5 cultivars × 5 replicates × 2 cultivation conditions (in soil or in hydroponics)].

### Sample Preparation

Harvested fruit were processed to obtain a homogenized tomato juice using a modified version of the method presented by Xiao et al. (2015). Briefly, 150 g of fruit per replicate were mixed to puree using a household blender (Waring Pro BB90E, Silva Homeline, Salzburg, Austria) that was sterilized and subsequently washed three times with sterilized water. Two 2-mL aliquots of the puree were transferred to sterile reaction tubes by using a disposable transfer pipette (VWR, Vienna, Austria) and directly frozen at −20°C for later DNA isolation. The remaining puree was then transferred to sterile tubes and centrifuged at 4°C and 14,000 × *g* for 15 min. The supernatant (fresh tomato juice) was recovered and further processed to determine volatile, sugar and acid concentrations.

### Measurement and Identification of Volatile Compounds in Tomato Fruit

For organic volatile analyses, 8 g of fresh tomato juice were combined with 1.5 g of sodium chloride, 100 μL EDTA/NaOH (1M, pH = 7.5) and 8 μL 1-octen-3-ol (100 mg/L) as internal standard. Samples and reagents were mixed thoroughly in a 20 mL headspace vial equipped with a PTFE/butyl septum (References: 5188-2753 and 5188-2759; Agilent Technologies, United States). Samples were conditioned by incubating each vial at 60°C for 15 min on a thermoblock stirring at 250 rpm. Subsequently, the sample headspace was exposed to the solid-phase micro extraction (SPME) fiber (65 μm PDMS/DVB, Reference: 57298-U; Supelco, United States) for further 50 min under the same conditions. Desorption of the analytes into the injector port of the gas chromatography (GC) apparatus was carried out at 270°C for 15 min with a 3:1 split ratio. Headspace conditioning, sampling and injection into the GC was performed with CombiPAL (CTC Analytics, Switzerland). Volatile compounds in the headspace of the tomato juice were separated on the Agilent 7890A GC. The gas flow was equally split after separation with a deans switch (Agilent Technologies) into two detectors, an Agilent 5975C mass selective (MS) detector for identification and a flame ionization detector (FID) for quantification. Chromatographic separation of the analytes took place in a VF-5ms (30 m × 250 μm × 0.25 μm) column (Agilent Technologies). The oven temperature was set to hold at 60°C for 6 min, followed by a ramp of 5°C/min to 280°C. Helium was used as gas carrier at a flow rate of 1 mL/min. The area of each peak was calculated by the ChemStation software (Agilent Technologies). To ensure that no peaks derived from previous runs or any instrumentation, blank runs with pure water were run every 12 samples. Identification of the volatile compounds was based on comparing mass-fragmented patterns with mass spectra in the NIST Database or with previously reported Kovats indices (KI) in the literature. Kovats indices were calculated by using retention data of n-alkanes (C7-C24) along with retention data of the analytes. Furthermore, the concentrations of the volatile compounds in each sample were quantified based on the peak area of the internal standard.

### Assessment of Sugar Content in Tomato Fruit

For the estimation of both sugar and acid content in the tomato fruit, the protocol presented by Agius et al. was employed (Agius et al., 2018). Briefly, 2 mL of freshly prepared tomato juice were filtered through a 0.22 μm membrane (Reference: 8-7066; Ahlstrom ReliaPrep, Finland). Subsequently, 400 μL of the filtrate were combined with 100 μL of both internal standards, namely tricarballylic acid (10 g/L) and lactose (100 g/L), as well as with 400 μL acetonitrile (ACN, 100%). The mixture was then centrifuged at 4°C and 14,000 × *g* for 5 min. Sugars and acids in the samples were separated by solid phase extraction (SPE) as indicated by the authors using one Supelclean™ LC-NH2 SPE 100 mg tube per sample (Reference: 504483, Sigma Aldrich). Sugar analyses were performed using high pressure liquid chromatography (HPLC) coupled with a diode array detector (DAD) by transferring 200 μL of sugar eluate to an autosampler vial together with 8 μL ACN 90%. Chromatographic separation of the sugars in the samples took place in a Shodex Asahipak NH2P-50 4E 125 mm × 4 mm column preceded by a Shodex Asahipak NH2P-50G 4A 10 mm × 4.6 mm precolumn (Showa Denko Europe, Germany). The column oven temperature was set to 40°C. 20 μL of the sample was injected. For elution, a mixture of 75% ACN and NaOH (pH = 12) was used at a flow rate of 1.1 ml/min. Quantification of sugar concentrations was based on the results obtained from a standard mix containing fructose, glucose and lactose at 5 concentration levels (Agius et al., 2018).

### Estimation of Acid Content in Tomato Fruit

Hundred microliters of eluate (see previous section) containing the acidic compounds of the tomato fruit was evaporated in a desiccator overnight. The next day, 120 μL of derivatization reagent (2M (trimethylsilyl) diazomethane in diethyl-ether together with 900 μL methanol) was added until the mixture remained yellow. The reaction was incubated at 25°C and 800 rpm for 5 min in a thermomixer. 5 μL of 1M acetic acid (in methanol) were added until the yellow color disappeared. The reactions were subsequently centrifuged at 14,000 × *g* for 5 min at room temperature. 50 μL of the reaction were transferred to an autosampler vial for analyses on GC-MS using the previously described column. Helium served as carrier gas at a flow rate of 1 mL/min. Injection was performed at 220°C in splitless mode. The temperature program was set to hold one minute at 45°C, then to increase at 15°C/min until 210°C and finally to reach 300°C at a rate of 30°C/min. The transfer line was operated at 280°C, the ion trap at 160°C and the manifold at 40°C. Quantification of acid concentrations was based on standard curves of citric and malic acids run at five concentrations (Agius et al., 2018).

### DNA Extraction

DNA was isolated from 0.5 mL tomato puree with the FastDNA^®^ SPIN Kit for Soil (MP Biomedicals, Solon, OH, United States) and following the manufacturers’ protocol using the beat-beating method. To test the efficacy of the protocol in extracting bacterial DNA from tomato puree, selected samples were mixed with 75 μL of thawed mock community suspension (ZymoBIOMICS™ Microbial Community Standard, Zymo Research, United States). In addition, DNA was isolated directly from the pure mock community using 75 μL to provide control DNA samples to test whether there are substances in the DNA extractions from tomato puree, which inhibit PCR. DNA (5 μL) was separated and visually tested for quality by electrophoresis (80 V) on 1% (w/v) agarose gels. In addition, DNA concentration was measured with a NanoDrop ND-1000 spectrophotometer (Thermo Fischer Scientific, DE, United States).

### 16S rRNA Library Preparation

To analyze the bacterial communities of tomato fruit the V5-V7 region of the 16S rRNA gene was amplified in a two-step PCR approach as described previously in comparable studies (Escobar Rodríguez et al., 2018). In the first step, PCR was carried out with the PCR primers 799f (5′- AACMGGATTAGATACCCKG -3′) and 1175r (5′- ACGGGCGGTGTGTRC -3′) in 25 μL reaction volume containing 5–10 ng DNA, 1 × KAPA buffer, 0.3 mM dNTPS, 0.3 μM forward and reverse primer each, 0.5 U KAPA polymerase (Kapa Biosystems, Boston, MA, United States) and PCR grade water. Amplifications were performed in a Thermocycler (peqSTAR 96x HPL, VWR International, Vienna, Austria) and cycling conditions were as follows: 95°C for 3 min, followed by 25 cycles of 30s at 95°C, 30s at 55°C and 30s at 72°C with a final extension step at 72°C for 5 min. Reactions were performed in triplicate and pooled. Pools were loaded onto a 2% agarose gel (w/v) in sterile-filtrated TAE (Biozym Biotech Trading, Vienna, Austria), and the bacterial 16S rRNA gene amplicons were separated from mitochondrial 18S rRNA gene amplicons by electrophoresis for 80 min at 110 V and excised from the gel using X-tracta Gel Extraction Tools (Sigma Aldrich, Vienna, Austria). The gel pieces were then placed inside the top part of a cut sterile filter tip placed inside a 2 mL Eppendorf tube and DNA collected by centrifugation at 10,000 rpm for 1 min in an Eppendorf™ 5424 Microcentrifuge (Eppendorf, Hamburg, Germany). Then, the filter tip was carefully removed with sterile tweezers, and 2 μL of the eluate were used for the second round of PCR with the primers 799f and 1175r that had specific indices (Supplementary Table 2) attached for sample recognition in sequencing. The concentration of PCR amplicons was estimated from the intensity of the amplicon bands on agarose gels with Image Lab™ Software 6.0 (BioRad, Hercules, CA, United States) using the bands of the DNA marker as a point of reference. For library preparation, 48 amplicons were pooled in equimolar amounts, and libraries purified with the Agencourt^®^ AMPure^®^ XP system (New England Biolabs, Ipswich, MA, United States). Total DNA in the library was quantified with Quant-iT™ PicoGreen^®^ following the instructions of the manufacturer in a plate reader (BioTek, Winooski, VT, United States). For sequencing, pooled and purified libraries were subjected to Illumina adapter ligation and sequencing using 2 × 250 bp MiSeq v2 sequencing at LGC Genomics (Berlin, Germany). Controls included in this analysis were: (1) the controls for DNA isolation efficacy described above, (2) the negative control for each PCR reaction, for which water was used instead of DNA and (3) pieces of the agarose gel used for the isolation of the bacterial amplicons.

### Raw Sequence Data Processing

For removing the Illumina inherent Phi X contamination raw reads were filtered with Bowtie2 v.2.3.4.3 (Langmead and Salzberg, 2012) and the overall quality was checked with FastQC v.0.11.8 (Andrews, 2010). Demultiplexing and stripping the adapters and primers was done with Cutadapt v.1.18 (Martin, 2018). The DADA2 v. 1.12.1 Bioconductor R package (Callahan et al., 2016) was then applied to quality filter, trim, denoise and merge paired-reads while also screening for chimeras. Using Metaxa2 v.2.2 (Bengtsson-Palme et al., 2015) unique Amplicon sequence variants (ASVs) stemming from the V5-V7 region of the 16S rRNA gene of bacteria and archaea were identified. Those were then assigned to taxa by using the RDP classifier (Wang et al., 2020) re-implemented in DADA2 against the SILVA SSU v.132 database (Quast et al., 2013) and finally an ASV count matric was generated for the statistical analysis. Prior to any further analyses, ASVs deriving from control samples were removed from the dataset.

### Bacterial Community Analyses

Representative bacterial communities in our samples were obtained by considering bacterial amplicon sequence variants (ASVs) with a relative abundance greater than 0.01%. A further filtering step consisted in extracting reproducibly-occurring ASVs (rASVs), namely those observed in at least three of five replicates. Alpha diversity values (total ASV count and Simpson’s Index) were calculated using the rtk R package (Saary et al., 2017) averaging the results of 999 iterations. The rarefaction minimum depth was 151 for reproducibly-occurring communities. For beta diversity analyses, a cumulative sum scaling (CSS) normalization was applied (Paulson et al., 2013). Indicator genera for all cultivars in each cultivation method was calculated fitting a multivariate generalized linear model to the rASV table (mvabund:manyglm).

### Statistical Analyses

Processed sequence and chemical data were analyzed in R v3.6.3 software. We investigated the role of cultivation method, tomato cultivar and interaction thereof (genotype x environment, GxE) in the concentrations of total volatile compounds, aroma-relevant volatiles and most abundant compounds, as well as in the amount of sugars (glucose and fructose) and acids (malic and citric) of the tomato fruit using analysis of variance (ANOVA). Volatile compound variation across samples was assessed employing principal component analysis (PCA) and visualized with factoextra:fviz_pca, depicting only those volatiles with a contribution greater than 10 in the loadings plot (Kassambara and Mundt, 2020). The effect of cultivation method, tomato cultivar, and GxE (genotype x environment) interaction in the diversity and composition of the microbial communities of the fruit was assessed using the Phyloseq (McMurdie and Holmes, 2013) and vegan (Oksanen et al., 2020) packages by testing with permutational multivariate analysis of variance (PERMANOVA, 9999 permutations). Pairwise comparisons among cultivars were conducted using the permwise.perm.manova of the RVAideMemoire package (Hervé, 2021), where *p* values were adjusted using false discovery rate controlling procedures. Unless otherwise indicated, statistical significance threshold was set at α = 0.05. Spearman correlation analyses between abundances of rASVs and absolute volatile, sugar and acid compound concentrations was conducted using microbiome:associate and depicted with the heat command considering significant adjusted *p-*values (Lahti et al., 2017).

### Nucleotide Sequence Accession Numbers

Sequence data are available in the NCBI SRA database under the BioProject number PRJNA513967.

## Results

### Fruit Volatile Profile Is Mainly a Function of the Tomato Cultivar, While Sugar and Acids Levels Depend on the Cultivation Method

**FIGURE 2:**
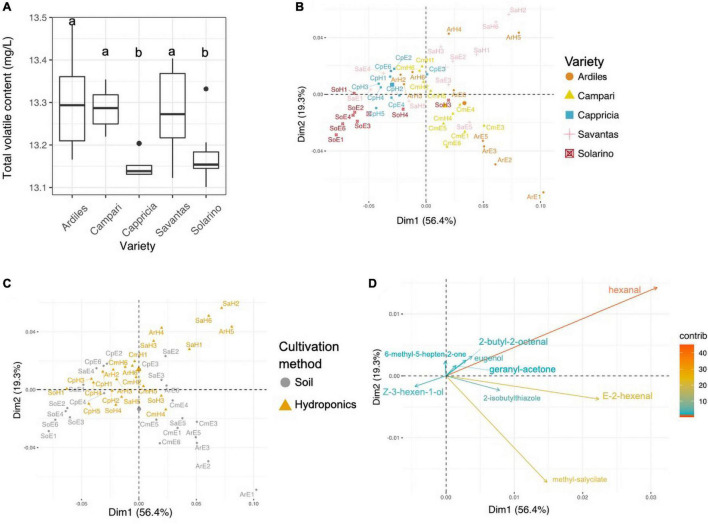
**(A)** Total concentration of organic volatile compounds (VOCs) in tomato fruit of five different cultivars expressed in mg/L. Letters over each box indicate members of homologous groups according to a Tukey HSD test for pairwise comparisons among tomato cultivars. **(B)** Principal component analyses (PCA) showing groupings of individual samples colored by tomato cultivar. **(C)** PCA with individual samples colored according to the employed cultivation method. **(D)** PCA loadings of VOCs with a contribution >10.

A total of 34 volatile organic compounds (VOCs) were detected by HS-SPME-GC-MS analysis of the tomato juice, including 13 aldehydes, 9 alcohols, four ketones, four esters and four other compounds (Table 1). Among all cultivars, VOCs with the highest measured concentrations included hexanal, *E*-2-hexenal, 2-isobutylthiazole and methyl salicylate. Sixteen of all detected compounds in this study either had log odor units > 0 (e.g., beta-damascenone), or were listed by Tieman and colleagues to be linked with overall liking by the consumer, sweetness perception and/or flavor intensity: isovaleraldehyde, benzaldehyde, *E-*2-heptenal, E-2-hexenal, nonyl-aldehyde, phenylacetaldehyde, guaiacol, 2-methylbutanol, eugenol, *Z*-3-hexen-1-ol, 6-methyl-5-hepten-2-one, beta-ionone, hexyl-acetate, 1-nitro-methylbutane, and 2-isobuthylthiazole (Tieman et al., 2012, 2017). There were no significant differences in total volatile amounts between tomatoes grown in soil and those from hydroponic cultures, while the cultivar and the GxE interaction significantly determined the total VOCs concentrations (Anova, F-values = 9.9 and 3.9; *p* < 0.001, respectively; Figure 2A). In fact, the concentrations of 20 individual VOCs were found to differ significantly among tomato cultivars, whereas only nine were affected by the cultivation method and GxE interactions (Table 1 and Supplementary Figure 1). PCA analysis of the VOC concentrations explained a total of 75.7% of the variance in the first two principal components (PC) and revealed clustering of samples belonging to the same fruit cultivar (Figure 2B). When grown in hydroponics, the cultivars ‘Savantas’ and ‘Ardiles’ were characterized mainly by their high hexanal contents together with less pronounced amounts of 2-butyl-octenal, eugenol and geranyl-acetone. The fruit of the soil-grown cultivars ‘Campari’ and ‘Ardiles’ were rich in methyl salycilate, *E*-2-hexenal and 2-isobutylthiazole. The cultivar ‘Solarino,’ soil-grown, however, showed characteristic amounts of 3-hexen-1-ol (Figures 2B,D). It was noteworthy that groupings within cultivation methods were not observed (Figure 2C). Prevalent volatiles among samples are displayed as variables and their loadings in Figures 2B–D, respectively as well as in Supplementary Figure 1.

**TABLE 1:** List of measured sugars and acids as well as volatile compounds (VOCs) detected in the headspace of juice made from tomato fruit of five different cultivars.

Compound class	Compound	Retention index	Odor description
Aldehydes (13)	Isovaleraldehyde 2,4-dimethyl-benzaldehyde ¤ 2-butyl-2-octenal ¤ 2-octenal ¤,* 4-propylbenzaldehyde ¤,*,§ Benzaldehyde ¤,§ Beta-cyclocitral Citral *E*-2-heptenal ¤ *E-*2-hexenal ¤,*,§ Hexanal ¤ Nonyl-aldehyde ¤,*,§ Phenylacetaldehyde ¤	1139 1378 1063 969 1230 1275 961 857 801 1082 1052	Malt Sweet, bitter almond Fatty, green Almond, burnt sugar Mint, herbal, tobacco Minty, citrus Fatty, almond Green, leafy Grass, fat Fat, citrus, green Sweet, honey, rose
Alcohols (9)	Guaiacol ¤ 2-methyl-6-hepten-1-ol ¤ 2-methyl-butanol 3-methyl-butanol 4-methyl-pentanol ¤ Eugenol ¤,*,§ Linalool Phenethylalcohol ¤,§ *Z-*3-hexen-1-ol ¤	1070 994 870 1367 1101 1120 828	Smoky, woody Malt, wine, onion Whiskey, malt, burnt Pungent Cinammon, clove, spice Lavender, lemon, rose Bitter, rose, spice Unripe banana, green, grass
Ketones (4)	6-methyl-5-hepten-2-one *,§ Beta-ionone * Beta-damascenone Geranyl acetone	989 1496 1360 1457	Citrus, mushroom Floral, violet, cedarwood Floral Sweet, floral, estery
Esters (4)	Propyl-acetate Ethyl-salicylate Hexyl-acetate * Methyl-salicylate ¤,*,§	1280 1002 1202	Celery, bitter Mint Fruity, green, banana Peppermint
Other (4)	1-nitro-3-methylbutane ¤ 1-nitro-2-phenylethane ¤ 2-isobutylthiazole ¤,§ Heptane ¤	904 1309 1040	Flower, spice Tomato, leafy, green
Sugars (2)	Total sugars ¤,* Glucose ¤,* Fructose ¤,*	–	–
Acids (2)	Total acids ¤,*,§ Citric acid ¤,*,§ Malic acid ¤,*	–	–
Total VOCs = 34	Total VOC concentrations ¤,§		

Underlined compound names indicate their presence in the list of compounds presented by Tieman et al. (2012, 2017), suggesting their relevance in the overall flavor intensity of the tomato fruit. Symbols (¤,*,§) indicate whether measured concentrations are function of the tomato cultivar, cultivation method, and interaction thereof (GxE), respectively. Excluding sugars and acids, compounds with no retention index were identified based solely on the comparison of their mass-fragmented patterns with mass spectra in the NIST Database. Odor descriptions of VOCs are adapted from Li et al. (2019) or were acquired from the chemistry database of the National Institutes of Health (NIH), PubChem.

**FIGURE 3:**
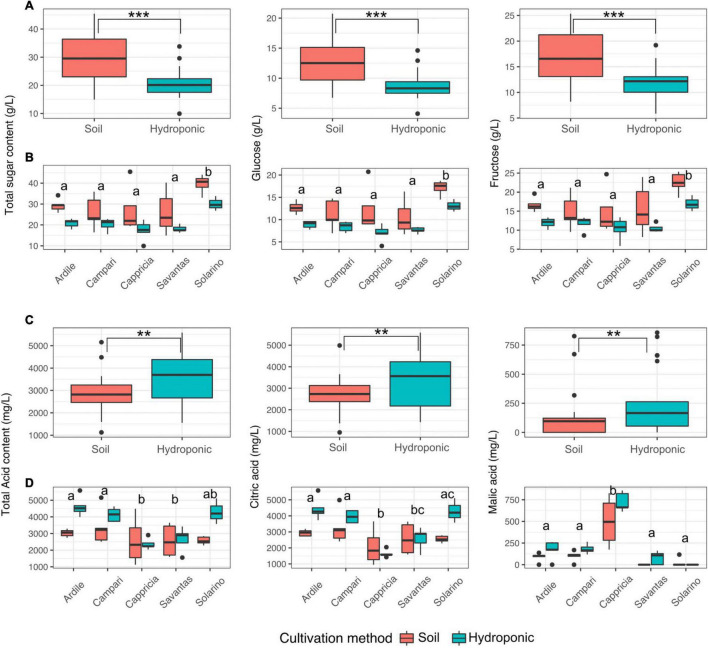
**(A)** Total sugar as well as glucose and fructose concentrations in juices prepared from tomatoes grown in soil and in hydroponics. **(B)** Sugar concentrations in juices of five different tomato cultivars grown in soil and hydroponics. **(C)** Total acid as well as malic and citric acid concentrations in juices prepared from tomatoes grown in soil and in hydroponics. **(D)** Acid concentrations in juices of five different tomato cultivars grown in soil and in hydroponics. Letters over each box indicate members of homologous groups according to a Tukey HSD test for pairwise comparisons among tomato cultivars. Significance values computed by ANOVA for each factor are indicated as (**) for *p* > 0.01 and (***) for *p* > 0.001.

Tomato fruit grown in soil showed significantly higher concentrations of glucose and fructose than those grown in hydroponics (Figure 3A). Fruit of the cultivar ‘Solarino’ had significantly more sugar than the other cultivars, with over 3.5% mean sugar content (Figure 3B). In contrast, amounts of the main organic acids were significantly higher in tomatoes grown in hydroponics (Figure 3C). Citric acid predominated in tomato fruit reaching up to 5 g/L and its concentration was significantly higher in the cultivars ‘Ardiles,’ ‘Campari’ and ‘Solarino’ grown in hydroponics. The content of malic acid, on the other hand, reached a maximum of 750 mg/L and was significantly higher in fruit of the cultivar ‘Cappricia’ (Figure 3D). Overall, the major contributors to the total and individual sugar content were mainly the cultivation method and secondly, the tomato cultivar (F-values = 24.2, 9.2; *p* < 0.001 respectively). All three factors significantly shaped the total acid concentration, with the main contributor being the cultivation method, followed by the cultivar and lastly, the GxE interaction (F-values = 12.05, 6.72 and 2.9, *p* < 0.01) respectively. Amounts of citric acid were mainly a function of the tomato cultivar, followed by the cultivation method and lastly the GxE interaction (F-values = 11.6, 10.8 and 3.9; *p* < 0.01, respectively), while the cultivar and the cultivation method were the main contributors to malic acid concentrations in the tomato fruit (F-values = 46.8, 8.3; *p* < 0.001, respectively) (Table 1).

### The Cultivation Method Affects the Bacterial Diversity in Tomato Fruit of Some Cultivars and Significantly Shapes the Community Structure

Sequencing of the V5 to V7 region of the 16S rRNA genes present in 50 tomato puree samples (5 cultivars × 5 replicates × 2 cultivation conditions resulted in a total of 983,497 high-quality merged reads, corresponding to an average of 19,669.94 ± 11,641.69 reads per sample (*n* = 50) and a mean length of 375 bp. ASVs with relative abundances greater than 0.01% were used for further analyses in R 3.6.3, resulting in a total of 851 ASVs.

**FIGURE 4:**
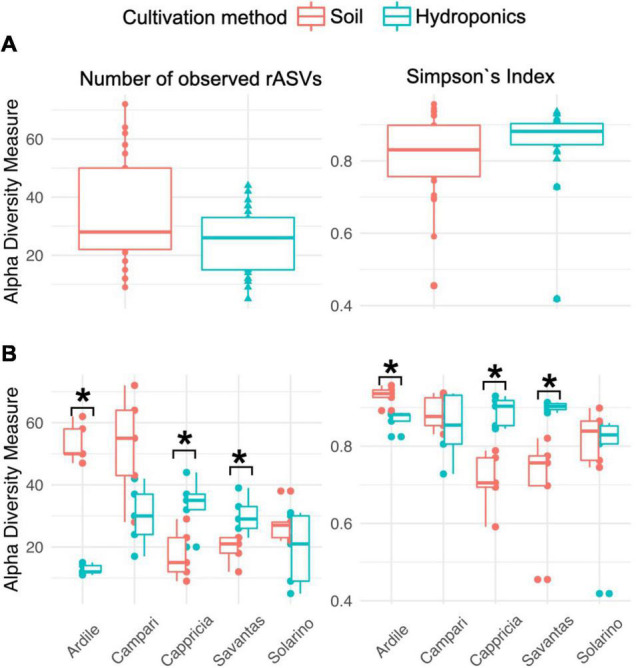
**(A)** Total observed rASVs and diversity (Simpsons Index) of tomato fruit grown in soil and in hydroponics. **(B)** Alpha diversity measures considering the effect of cultivation method in each tomato cultivar. Significance values computed by ANOVA are indicated as (*) for *p* > 0.05.

**FIGURE 5:**
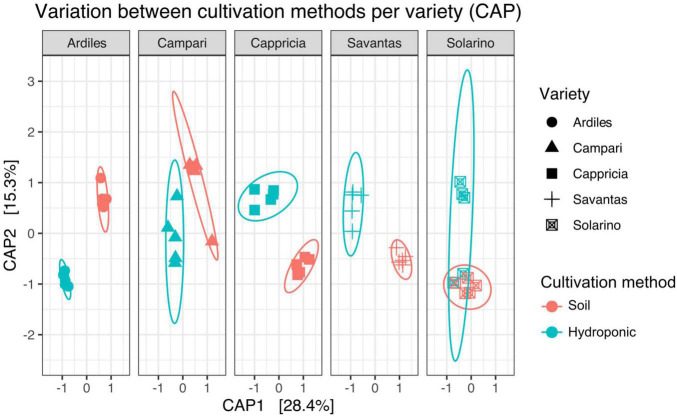
Variation across samples of both cultivation methods is visible for each tomato cultivar (CAP).

We investigated the role of both, cultivation method and tomato cultivar, in the diversity and composition of the reproducibly occurring bacterial communities of the fruit. We could not ascertain any significant differences in the numbers of total rASVs or diversity between fruit grown in soil and those from hydroponic cultures (Figure 4A). However, there were significant effects observed in the GxE interaction [F = 21.52, Pr (> F) = 0.0001]: numbers of identified rASVs were significantly higher in hydroponic “Cappricia” and “Savantas” tomatoes compared to their soil-derived counterparts, whereas soil cultivated “Ardiles” fruit harbored more bacterial rASVs than those that were grown in hydroponics. The same trend was observed in the Simpson indices (Figure 4B). In contrast, no significant differences in alpha diversity among tomato cultivars were observed. In terms of beta diversity, the cultivation method, tomato cultivar and the interaction thereof significantly shaped the bacterial community composition of the tomato fruit (Table 2). The largest amount of variation among samples was observed in the communities of hydroponic “Solarino” fruit as indicated by the scattering of samples across the second axis of the constrained ordination plot (CAP) depicted in Figure 5. Moreover, the effect of the cultivar on the bacterial community structures of the fruit was significantly stronger in soil-grown tomatoes than from those of hydroponic cultures: while communities of all soil-derived tomatoes differed significantly from each other and from their hydroponic counterparts, no significant differences were identified among hydroponic fruit of “Solarino” and the rest of cultivars, as well as between “Cappricia” and “Savantas” (Supplementary Table 3).

**TABLE 2:** Roles of cultivation method and variety in the bacterial community structures of tomato fruits.

	F. Model	R2	Pr(>F)
Cultivation method	29.81	0.24	1e-04 (***)
Variety	4.96	0.16	1e-04 (***)
Cultivation method:Variety	8.57	0.27	1e-04 (***)

Significance values computed by ANOVA for each factor are indicated as (***) for p > 0.001.

### The Taxonomic Composition of Bacterial Communities Colonizing Tomato Fruit From Cultivation in Soil and in Hydroponics

**FIGURE 6:**
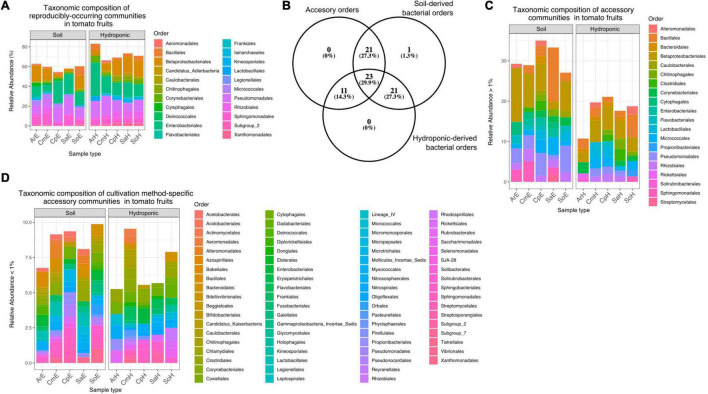
**(A)** Relative abundance of core orders in soil- and hydroponic grown tomato fruit. rASVs represent a higher portion of the overall communities in hydroponic tomatoes (65–82%) as compared to soil derived fruit (∼60%). Reproducibly occurring communities of hydroponic tomatoes lack member rASVs from Flavobacteriales and Kineosporiales. **(B)** Number of accessory bacterial orders (accessory) that are specific to soil-grown tomatoes or hydroponic-grown fruit. **(C)** Abundance of 20 predominant accessory bacterial orders detected in tomato fruit. None of these orders were specific to any cultivation method, but rather common among them. **(D)** Accessory bacterial orders that were specific to each cultivation method occurred in low relative abundance.

Overall, a total of 851 ASVs were classified among 77 bacterial orders of 18 classes, with an average of 17.02 ASVs per sample (Supplementary Data 1). ASVs classified as Proteobacteria were predominant among tomato fruit, with a relative abundance of over 30% of total reads, followed by Firmicutes, Actinobacteria and Bacteroidetes with around 5% prevalence each. Most represented were orders of the Gammaproteobacteria (Enterobacteriales and Pseudomonadales, Betaproteobacteriales), and Alphaproteobacteria (Rhizobiales and Sphingomonadales). At the genus level, *Pseudomonas, Burkholderia-Caballeronia-Paraburkholderia, Raoultella, Sphingomonas, Massilia* and *Paenibacillus* were among the most abundant taxa detected in the tomato fruit in this study (Supplementary Figure 2). In order to identify representative bacterial communities (“core”) within tomatoes from both cultivation methods, we extracted reproducibly occurring ASVs (rASVs) from the data set for further investigation. One hundred and twenty-seven (127) rASVs occurred in at least 3 of 5 replicates and were classified among 22 bacterial orders. These reproducibly occurring communities represented a higher portion of the overall bacterial abundance in hydroponic fruit (up to 80%) compared to those from soils (around 60%). Differences between reproducibly occurring communities of tomatoes from both cultivation methods were minimal in taxonomic composition but evident through the lack of members of the Flavobacteriales and Kineosporiales in hydroponic tomatoes (Figure 6A). Furthermore, of the remaining 55 “accessory” bacterial orders (i.e., which did not occur in a reproducible fashion), 21 were identified exclusively in soil-grown tomatoes and eleven were specific to fruit from plants grown in hydroponics (Figure 6B). Accessory bacterial orders that were specific to any of the cultivation methods occurred in low relative abundances (Figure 6D). Twenty-three orders occurred in fruit grown in both soil and hydroponics (Supplementary Data 1 and Figures 6C,D).

### Indicator Genera of Cultivation Method Correlate With the Abundance of Specific Volatile and Non-volatile Compounds

**FIGURE 7:**
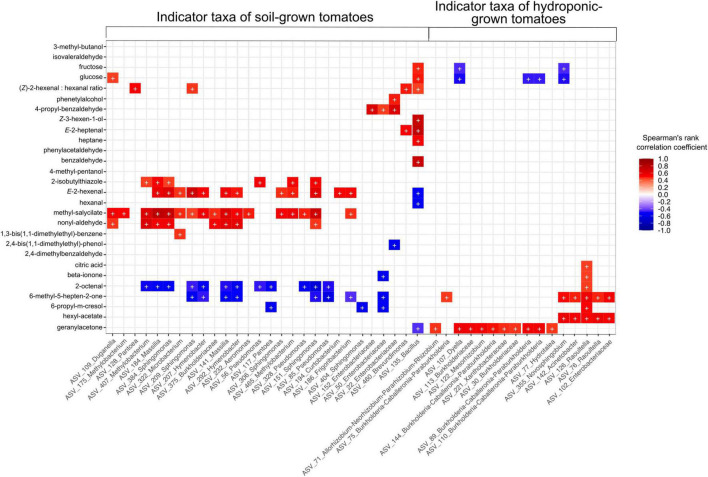
Relative abundance of 46 indicator rASVs for the Cultivation soil × Cultivar (GxE) interaction correlate with concentrations of at least one aroma or flavor compound in this study. Symbols in cells (+) indicate significant correlations (fdr adjusted *p* > 0.05). Colors in cells with non-significant correlations have been removed for simplification.

Relative abundances of 99 rASVs correlated with the concentration of at least one volatile or non-volatile compound detected in this study. Among these, 46 rASVs belonging to 23 bacterial genera were identified as indicator species in at least one sample type (cultivar/cultivation method combinations) (Supplementary Figure 3A). Indicator rASVs of soil-grown tomatoes included members of *Pseudomonas, Pantoea, Sphingomonas, Massilia, Methylobacterium* and *Hymenobacter*, whose relative abundances correlated significantly with higher concentrations of methyl salicylate, nonyl aldehyde, 2-isobutylthiazole, *E-*2-hexenal and lower levels of 2-octenal and 6-methyl-5-hepten-2-one. Furthermore, soil derived “Solarino” tomatoes were characterized by higher abundances of a *Bacillus* (rASV_135) which was depleted in their hydroponic counterparts and not detectable in other tomato cultivars. This *Bacillus* rASV_135 correlated with higher abundances of fructose and glucose, as well as increased concentration of important aroma volatiles such as *Z-*3-hexen-1-ol, *E-*2-heptenal and benzaldehyde. Moreover, its abundance negatively correlated with lower concentrations of geranylacetone, *E-*2-hexenal and hexanal. On the other hand, indicator communities of tomatoes grown in hydroponics were represented by members of *Burkholderia-Caballeronia-Paraburkholderia*, *Dyella, Mesorhizobium, Raoultella, Acinetobacter* and *Novosphingobium.* The relative abundance of the former three genera correlated with higher levels of geranylacetone while the latter three correlated with concentrations of beta-ionone, 6-methyl-5-hepten-2-one and hexylacetate. Furthermore, relative abundances of indicator genera from hydroponic tomatoes correlated with decreased sugar levels. Moreover, an rASV classified as *Raoultella* (rASV_126) was characteristic of “Savantas,” “Campari” and “Cappricia” tomatoes grown in hydroponics and correlated positively with citric acid concentrations (Figure 7 and Supplementary Figure 3B).

## Discussion

### Aroma Profiles of Fruits Are Characteristic of the Cultivar Whereas the Cultivation Method Affects Amounts of Flavor Compounds

In this study, we asked whether tomatoes from different cultivars grown in soil or in a hydroponic system differ for their emission or content of volatiles, acids, and sugars, and whether such changes in chemical composition may be linked to the bacterial communities in tomato fruit. Volatile profiles in this study were mainly shaped by the tomato cultivar, rather than by the cultivation method. Volatiles related to flavor are derived from essential compounds like fatty acids, amino acids and color carotenoids (Goff and Klee, 2006), which vary in amounts and bioavailability among fruits of wild species and heirlooms and also across commercial hybrids (Rambla et al., 2014). This variability has been attributed to modern breeding processes, which led to the loss of superior alleles for volatiles associated with good aroma (Tieman et al., 2017).

In contrast, the levels of acids and sugars were mainly affected by the cultivation method in this study: soil grown tomatoes showed significantly higher levels of dominant sugars like glucose and fructose, whereas hydroponic tomatoes accumulated higher amounts of citric and malic acids. Numerous studies have shown how the cultivation conditions strongly affect tomato yield, fruit morphology and biochemistry (Kuti and Konuru, 2005; Ortiz et al., 2007; Roselló et al., 2011; Adalid et al., 2012; Panthee et al., 2013; Figàs et al., 2015, 2018): regarding fruit quality, tomato cultivars grown in greenhouse conditions were richer in sugars and antioxidants but poorer in lycopene and ß-carotene than tomatoes from open fields (Kuti and Konuru, 2005; Beckles, 2012; Figàs et al., 2015, 2018), but no difference was found for organic acids under these conditions (Galiana-Balaguer et al., 2006; Figàs et al., 2018). Similarly, grapevine cultivars grown under different conditions result in berries and wines with different biochemical composition and sensory characteristics (Pereira et al., 2005; Son et al., 2009; Robinson et al., 2012).

Several studies have shown that moderate stress conditions may improve tomato fruit quality through increased concentration of flavor compounds in some cultivars including sugars, organic acids, carotenoids and phenolic compounds (Zheng et al., 2013; Albert et al., 2016a,b). While those studies focused on the effects of abiotic stresses during cultivation (e.g., water deficit, salinity and/or heat) on the metabolite production of tomato fruit (reviewed by Quinet et al., 2019), it is possible that tomato plants grown in soil undergo intensified biotic stresses compared to those from hydroponic cultures, which could ultimately lead to changes in the fruit chemistry. Soils contain an extraordinary number and diversity of microorganisms that may interact directly or indirectly with the roots of a nearby plant, e.g., by inducing immune responses and/or altering metabolic pathways (Zamioudis and Pieterse, 2011; Hardoim et al., 2015). In fact, we found that soil grown “Ardiles” and “Campari” tomatoes were characterized by high levels of methyl salicylate, a volatile compound from the phenylpropanoid metabolic pathway known to be involved in plant stress responses (Shulaev et al., 1997; Seskar et al., 1998). This goes hand-in-hand with the findings of Chialva and colleagues, who observed that the microbiota rather than the chemico-physical properties of native soils were crucial in eliciting stress responses in some tomato cultivars, including phenylpropanoid metabolism (Chialva et al., 2018). As for organoleptic perception, methyl salicylate has been described to possess “medicinal” or “peppermint” notes (Zanor et al., 2009), and although it is one of the major contributors to overall tomato flavor, it has been correlated with poor flavor acceptability at higher levels (Vogel et al., 2010). Therefore, biotic stresses related to the microbial complexity of the cultivation substrate may contribute to the altered production of sugars, acids and volatiles in a cultivar-specific manner.

### Bacterial Diversity and Community Composition of Tomato Fruits Is Shaped by the Cultivation Method in a Cultivar-Dependent Manner

We further analyzed the bacterial community composition of the tomato fruit and observed no significant differences in total numbers of rASVs nor in alpha diversity measures (Simpson’s index) between tomatoes grown in soil or in hydroponics. However, when we considered the tomato cultivar, the role of the cultivation method in alpha diversity became evident for ‘Ardiles,’ ‘Cappricia’ and ‘Savantas’ tomatoes, while no effect was found in ‘Solarino’ and ‘Campari.’

Plant cultivars are often differently affected by the environment. This phenomenon is known as genotype x environment (GxE) interaction. The tomato plants in this study were grown in controlled hydroponic culture with relatively limited stimuli and environmental variations and, on the other hand, in pots filled with soil inside a wire-house with natural fluctuations in temperature, precipitation and interactions with (micro-)organisms below- and above-ground, providing a myriad of stimuli. It is therefore possible that each of the tomato cultivars reacted differently to these cultivation-dependent stimuli, resulting in fruit with divergent chemical characteristics. These variations in chemical environment within fruit may ultimately provide conditions that support distinct bacterial communities in tomatoes in each cultivar.

Indeed, we observed differences in the composition of the bacterial communities colonizing tomato fruit among the different cultivars and between the cultivation methods. Tomato fruit from all cultivars in this study did not only differ from each other in size (Figure 1), but also in pericarp consistency, locule number and, consequently, water content (not shown). Different tissues of tomato and apple fruit were found to be colonized by distinct bacterial communities (Dong et al., 2019; Wassermann et al., 2019). Therefore, cultivar-specific fruit characteristics like morphology, nutritional availability within the carposphere, size and biomass are likely to be responsible for the observed differences in microbial diversity among cultivars.

In contrast to soils, hydroponic cropping ecosystems are characterized by poor microbial complexities as the microorganisms in these environments derive mainly from plants or seeds, water, insects and personnel (Strayer, 1994). Moreover, plants harbor microbial communities inside, and on every organ. The assembly of these communities depends, among other factors, on the microbial availability in their immediate surroundings. Accordingly, community composition of soil-grown tomatoes of all cultivars differed significantly from their hydroponic counterparts. Interestingly, tomatoes from soil cultures developed “signature” bacterial communities, which were unique for each cultivar and were not observed in hydroponic fruit. Due to their microbial diversity and abundance, soils are considered the primary force driving microbial communities of roots and, albeit less pronounced, those of upper plant organs such as fruit (Zarraonaindia et al., 2015; Müller et al., 2016). Many plant-associated bacteria derive from the rhizosphere environment and may colonize the endospheres by mechanisms that are analogous for both soil and hydroponic cultures: bacteria are attracted to the rhizosphere environment by root exudates and rhizodeposits (Compant et al., 2010). From the roots, bacteria can also migrate to aerial compartments through the xylem of intercellular spaces (Hardoim et al., 2015). Bacteria in fruit may also derive from above-ground sources, like aerosols, pollen or insects (Compant et al., 2011; Glassner et al., 2017; Allard et al., 2018). Root exudate composition shapes microbial communities and have been shown to vary in amount and chemical composition depending on the plant genotype, health and developmental stage (Sasse et al., 2018). Furthermore, irrespective of the point of entry into the plant, microbial colonizers elicit immune responses that are also highly dependent on the above-mentioned host characteristics (Chialva et al., 2018), which shape the community composition by facilitating colonization of some microbial species and discriminating potential pathogens (Brader et al., 2017). Therefore, it is not surprising that fruit of different tomato cultivars harbor distinct microbiotas when provided with a highly diverse substrate like soil.

### Bacterial Taxa Correlate With Flavor and Aroma Chemistry of Tomato Fruits

We further aimed to address whether the differences in flavor compounds observed among tomato fruit grown under both cultivation methods are attributable to the abundances of specific members within the fruit microbiota. Indeed, we observed significant correlations among indicator species of soil-grown tomatoes, which included members of genera that have been previously found to be associated with fruit of tomatoes and other plants such as *Bacillus, Pseudomonas, Sphingomonas, Massilia*, and *Methylobacterium* (Verginer et al., 2010; Ottesen et al., 2013; Gilbert et al., 2014; Allard et al., 2018). Abundances of these taxa were associated with higher concentrations of volatiles described to contribute to the characteristic pungent, green, and leafy notes of the tomato fruit, namely methyl salicylate, nonyl aldehyde, 2-isobutylthiazole, *E-*2-hexenal (Klee, 2010; Vogel et al., 2010; Wang et al., 2015; Li et al., 2019). In contrast, indicator species of hydroponic tomatoes included members of *Burkholderia-Caballeronia-Paraburkholderia*, as well as genera that have been less described in fruit, namely *Dyella, Mesorhizobium, Raoultella, Acinetobacter* and *Novosphingobium.* Indicator members of these taxa correlated negatively with sugar levels and positively with levels of citric acid and apocarotenoid volatiles such as geranyl acetone, beta-ionone and 6-methyl-5-hepten-2-one. Apocarotenoid volatiles are products of the oxidative cleavage of various linear and cyclic carotenoids such as lycopene and beta carotene, which fulfill functions as colorants and nutrients in tomatoes. Volatiles derived from carotenoids have generally been described as having fruity and/or floral attributes and contribute to the sweetness perception of tomato fruit (Vogel et al., 2010). Also, reduced levels of beta ionone and 6-methyl-5-hepten-2-one have been associated with poor flavor in tomatoes (Tieman et al., 2017). Therefore, in terms of organoleptic perception, it is possible that the loss of sweetness related to the decreased sugar content observed in hydroponic tomatoes may be compensated to an extent by an increase in the amounts of apocarotenoid volatiles, resulting in an acceptable product for the consumer.

Furthermore, we observed that the relative abundance of rASV_135, classified as *Bacillus* (detected in all biological replicates of soil “Solarino” tomatoes but not in other samples) correlated with higher levels of both sugars and important aroma compounds. It is well known that *Bacillus* spp. are prominent soil inhabitants and typical plant colonizers (Yuan et al., 2015; Amaresan et al., 2019). He and colleagues showed that several *Bacillus* strains enhanced yield, growth and nutrient uptake of tomato plants when inoculated in the soil (He et al., 2019). A recent study also detected and isolated various *Bacillus* strains from tomato seeds which displayed multiple positive traits for their hosts including siderophore production and ACC-deaminase activity (Bergna et al., 2018). Accordingly, the authors proposed the seeds as carriers of bacteria into subsequent generations of tomato plants providing beneficial traits. To address the possible origins of potential modulators of tomato flavor such as rASV_135, we looked into bacterial communities of both irrigation water and tomato seedlings germinated under sterile conditions (unpublished). While the irrigation water had the highest overlap of ASVs with fruit in all cultivars, rASV_135 occurred persistently within “Solarino” seedlings, together with other seedling-derived rASVs of *Pantoea, Methylobacterium* and *Burkholderia-Caballeronia* and *Paraburkholderia*, which also correlated with abundance of tomato flavor compounds (Supplementary Data 2). Therefore, it is possible that within the fruit, we have detected seed-derived *Bacilli* and other microorganisms which may thrive when the seed is cultivated under certain conditions (e.g., soil *vs.* hydroponics). Although the correlation between the abundance of microbiota members like rASV_135 and the accumulation of flavor-relevant compounds in soil cultivated “Solarino” tomatoes does not necessarily imply causation, the potential role of fruit associated bacteria in the flavor profiles of tomato merits further investigation.

Summarizing, bacterial populations within fruit are shaped by the cultivation method and correlated with the concentrations of chemical compounds known for their implications in the organoleptic quality in these commodities. The tomato cultivar was indeed a key factor shaping the bacterial communities within fruit only when plants were grown in soil, indicating that the microbial diversity of the substrate influences the fruit microbiota. It remains unclear to what extent the correlations between microbial indicator taxa and aroma relevant compounds reflect causal associations. Microbial-mediated modulation of the tomato fruit flavor may occur either directly through the release of metabolites in the fruit or indirectly by enzymatic activities following cell disruption (Bokulich et al., 2016). Further studies investigating the function of the active members of the tomato fruit microbiota will help to disentangle the plant response to key bacterial populations in tomato fruit and are required to understand the role of plant-microbe interactions in fruit flavor. Moreover, the exact pathways involved in the accumulation of aroma-relevant volatiles and their precursors still demand investigation as tomato flavor is not solely based on the amounts of the single volatiles and non-volatile compounds but is rather shaped by the interactions among them, enhancing the perception of some traits while canceling others.

## Data Availability Statement

The datasets presented in this study can be found in online repositories. The names of the repository/repositories and accession number(s) can be found below: NCBI BioProject – PRJNA782607.

## Author Contributions

CE established the chemical analyses of flavor compounds, and mainly contributed in analyses of chemical and microbial data and in the composition of the manuscript. FB and PU contributed in sampling, sample preparation, sequencing and microbial data analyses. MG was mainly involved in tomato plant cultivation and sampling. LB performed analyses of sugars and acids. LA performed the raw data processing of the microbial sequences. JN and BM worked on experimental conception and design, data analyses and discussion of results. All authors contributed to the article and approved the submitted version.

## Conflict of Interest

The authors declare that the research was conducted in the absence of any commercial or financial relationships that could be construed as a potential conflict of interest.

## Publisher’s Note

All claims expressed in this article are solely those of the authors and do not necessarily represent those of their affiliated organizations, or those of the publisher, the editors and the reviewers. Any product that may be evaluated in this article, or claim that may be made by its manufacturer, is not guaranteed or endorsed by the publisher.
