# Supplementary material for: The Bacterial Microbiome of the Tomato Fruit Is Highly Dependent on the Cultivation Approach and Correlates With Flavor Chemistry
Source: Front Plant Sci. 2021 Dec 24;12:775722. doi: 10.3389/fpls.2021.775722 (PMC8740158; doi:10.3389/fpls.2021.775722)
Supplement: Supplementary file 1 [file Table_1.docx]

**Supplementary Table 2:** List of PCR primers for the second round of amplification. Nucleotides in red indicate the primer-specific indices.

| **Primer name** | **Oligo Sequence (5' to 3')** |
| --- | --- |
| 799F_1 | ACAACCAGTTAACMGGATTAGATACCCKG |
| 799F_2 | NAACAGACCTTAACMGGATTAGATACCCKG |
| 799F_3 | NNACAAGGTCTTAACMGGATTAGATACCCKG |
| 799F_4 | NNNAAGTCTTCGTAACMGGATTAGATACCCKG |
| 799F_5 | ACATGAGGTTAACMGGATTAGATACCCKG |
| 799F_6 | NAAGCTCACTTAACMGGATTAGATACCCKG |
| 799F_7 | NNACGATACGTTAACMGGATTAGATACCCKG |
| 799F_8 | NNNAATGCGCTATAACMGGATTAGATACCCKG |
| 799F_9 | ACCTCATCTTAACMGGATTAGATACCCKG |
| 799F_10 | NAACAGCTCATAACMGGATTAGATACCCKG |
| 799F_11 | NNACTGTTGACTAACMGGATTAGATACCCKG |
| 799F_12 | NNNAGAGTTGCTTAACMGGATTAGATACCCKG |
| 799F_13 | ACCTTGACATAACMGGATTAGATACCCKG |
| 799F_14 | NACAACTGTGTAACMGGATTAGATACCCKG |
| 799F_15 | NNACTTAGCACTAACMGGATTAGATACCCKG |
| 799F_16 | NNNAATACGACCTAACMGGATTAGATACCCKG |
| 799F_17 | ACGATCGTATAACMGGATTAGATACCCKG |
| 799F_18 | NACCAATCAGTAACMGGATTAGATACCCKG |
| 799F_19 | NNAGGACTTGTTAACMGGATTAGATACCCKG |
| 799F_20 | NNNAGCTGAATCTAACMGGATTAGATACCCKG |
| 799F_21 | ACTCACTGTTAACMGGATTAGATACCCKG |
| 799F_22 | NAGAGCAATGTAACMGGATTAGATACCCKG |
| 799F_23 | NNAGTCATCCTTAACMGGATTAGATACCCKG |
| 799F_24 | NNNAGTAGCCTATAACMGGATTAGATACCCKG |
| 799F_25 | AGATAGCGATAACMGGATTAGATACCCKG |
| 799F_26 | NAACGGAACATAACMGGATTAGATACCCKG |
| 799F_27 | NNATATAGCCGTAACMGGATTAGATACCCKG |
| 799F_28 | NNNAGTGAACTCTAACMGGATTAGATACCCKG |
| 799F_29 | ATACGGACTTAACMGGATTAGATACCCKG |
| 799F_30 | NAGCTCCTTATAACMGGATTAGATACCCKG |
| 799F_31 | NNATGCGCATATAACMGGATTAGATACCCKG |
| 799F_32 | NNNAGTGCTTCATAACMGGATTAGATACCCKG |
| 799F_33 | ATCACCATGTAACMGGATTAGATACCCKG |
| 799F_34 | NAGCTTCAGTTAACMGGATTAGATACCCKG |
| 799F_35 | NNATGTACTGGTAACMGGATTAGATACCCKG |
| 799F_36 | NNNATCGGTAGTTAACMGGATTAGATACCCKG |
| 799F_37 | ATCGAACCTTAACMGGATTAGATACCCKG |
| 799F_38 | NAGGTACAACTAACMGGATTAGATACCCKG |
| 799F_39 | NNATTGGAGTGTAACMGGATTAGATACCCKG |
| 799F_40 | NNNAGACATTCCTAACMGGATTAGATACCCKG |
| 799F_41 | ATGCAACACTAACMGGATTAGATACCCKG |
| 799F_42 | NAGGTGTGTTTAACMGGATTAGATACCCKG |
| 799F_43 | ATGGTAACGTAACMGGATTAGATACCCKG |
| 799F_44 | NAGTCGATACTAACMGGATTAGATACCCKG |
| 799F_45 | ATTCACCTGTAACMGGATTAGATACCCKG |
| 799F_46 | NAGTTGAGCATAACMGGATTAGATACCCKG |
| 799F_47 | ATTGACACCTAACMGGATTAGATACCCKG |
| 799F_48 | NATACGTTGCTAACMGGATTAGATACCCKG |
| 1175R_1 | ACAACCAGTTACGTCRTCCCCDCCTTCCT |
| 1175R_2 | NAACAGACCTTACGTCRTCCCCDCCTTCCT |
| 1175R_3 | NNACAAGGTCTTACGTCRTCCCCDCCTTCCT |
| 1175R_4 | NNNAAGTCTTCGTACGTCRTCCCCDCCTTCCT |
| 1175R_5 | ACATGAGGTTACGTCRTCCCCDCCTTCCT |
| 1175R_6 | NAAGCTCACTTACGTCRTCCCCDCCTTCCT |
| 1175R_7 | NNACGATACGTTACGTCRTCCCCDCCTTCCT |
| 1175R_8 | NNNAATGCGCTATACGTCRTCCCCDCCTTCCT |
| 1175R_9 | ACCTCATCTTACGTCRTCCCCDCCTTCCT |
| 1175R_10 | NAACAGCTCATACGTCRTCCCCDCCTTCCT |
| 1175R_11 | NNACTGTTGACTACGTCRTCCCCDCCTTCCT |
| 1175R_12 | NNNAGAGTTGCTTACGTCRTCCCCDCCTTCCT |
| 1175R_13 | ACCTTGACATACGTCRTCCCCDCCTTCCT |
| 1175R_14 | NACAACTGTGTACGTCRTCCCCDCCTTCCT |
| 1175R_15 | NNACTTAGCACTACGTCRTCCCCDCCTTCCT |
| 1175R_16 | NNNAATACGACCTACGTCRTCCCCDCCTTCCT |
| 1175R_17 | ACGATCGTATACGTCRTCCCCDCCTTCCT |
| 1175R_18 | NACCAATCAGTACGTCRTCCCCDCCTTCCT |
| 1175R_19 | NNAGGACTTGTTACGTCRTCCCCDCCTTCCT |
| 1175R_20 | NNNAGCTGAATCTACGTCRTCCCCDCCTTCCT |
| 1175R_21 | ACTCACTGTTACGTCRTCCCCDCCTTCCT |
| 1175R_22 | NAGAGCAATGTACGTCRTCCCCDCCTTCCT |
| 1175R_23 | NNAGTCATCCTTACGTCRTCCCCDCCTTCCT |
| 1175R_24 | NNNAGTAGCCTATACGTCRTCCCCDCCTTCCT |
| 1175R_25 | AGATAGCGATACGTCRTCCCCDCCTTCCT |
| 1175R_26 | NAACGGAACATACGTCRTCCCCDCCTTCCT |
| 1175R_27 | NNATATAGCCGTACGTCRTCCCCDCCTTCCT |
| 1175R_28 | NNNAGTGAACTCTACGTCRTCCCCDCCTTCCT |
| 1175R_29 | ATACGGACTTACGTCRTCCCCDCCTTCCT |
| 1175R_30 | NAGCTCCTTATACGTCRTCCCCDCCTTCCT |
| 1175R_31 | NNATGCGCATATACGTCRTCCCCDCCTTCCT |
| 1175R_32 | NNNAGTGCTTCATACGTCRTCCCCDCCTTCCT |
| 1175R_33 | ATCACCATGTACGTCRTCCCCDCCTTCCT |
| 1175R_34 | NAGCTTCAGTTACGTCRTCCCCDCCTTCCT |
| 1175R_35 | NNATGTACTGGTACGTCRTCCCCDCCTTCCT |
| 1175R_36 | NNNATCGGTAGTTACGTCRTCCCCDCCTTCCT |
| 1175R_37 | ATCGAACCTTACGTCRTCCCCDCCTTCCT |
| 1175R_38 | NAGGTACAACTACGTCRTCCCCDCCTTCCT |
| 1175R_39 | NNATTGGAGTGTACGTCRTCCCCDCCTTCCT |
| 1175R_40 | NNNAGACATTCCTACGTCRTCCCCDCCTTCCT |
| 1175R_41 | ATGCAACACTACGTCRTCCCCDCCTTCCT |
| 1175R_42 | NAGGTGTGTTTACGTCRTCCCCDCCTTCCT |
| 1175R_43 | ATGGTAACGTACGTCRTCCCCDCCTTCCT |
| 1175R_44 | NAGTCGATACTACGTCRTCCCCDCCTTCCT |
| 1175R_45 | ATTCACCTGTACGTCRTCCCCDCCTTCCT |
| 1175R_46 | NAGTTGAGCATACGTCRTCCCCDCCTTCCT |
| 1175R_47 | ATTGACACCTACGTCRTCCCCDCCTTCCT |
| 1175R_48 | NATACGTTGCTACGTCRTCCCCDCCTTCCT |
