# Supplementary material for: The Bacterial Microbiome of the Tomato Fruit Is Highly Dependent on the Cultivation Approach and Correlates With Flavor Chemistry
Source: Front Plant Sci. 2021 Dec 24;12:775722. doi: 10.3389/fpls.2021.775722 (PMC8740158; doi:10.3389/fpls.2021.775722)

**Supplementary Figure 1**: Concentrations of VOCs in fruits of five different tomato varieties cultivated in soil and hydroponically. The 17 VOCs shown are either relevant in the flavor intensity of tomatoes (according to Tieman et. al, 2017, name underlined) or were present in concentrations greater than 0.01 mg/L in at least one variety. Symbols indicate whether a compound is either significantly different among varieties (¤) and/or were affected by the cultivation method (*).


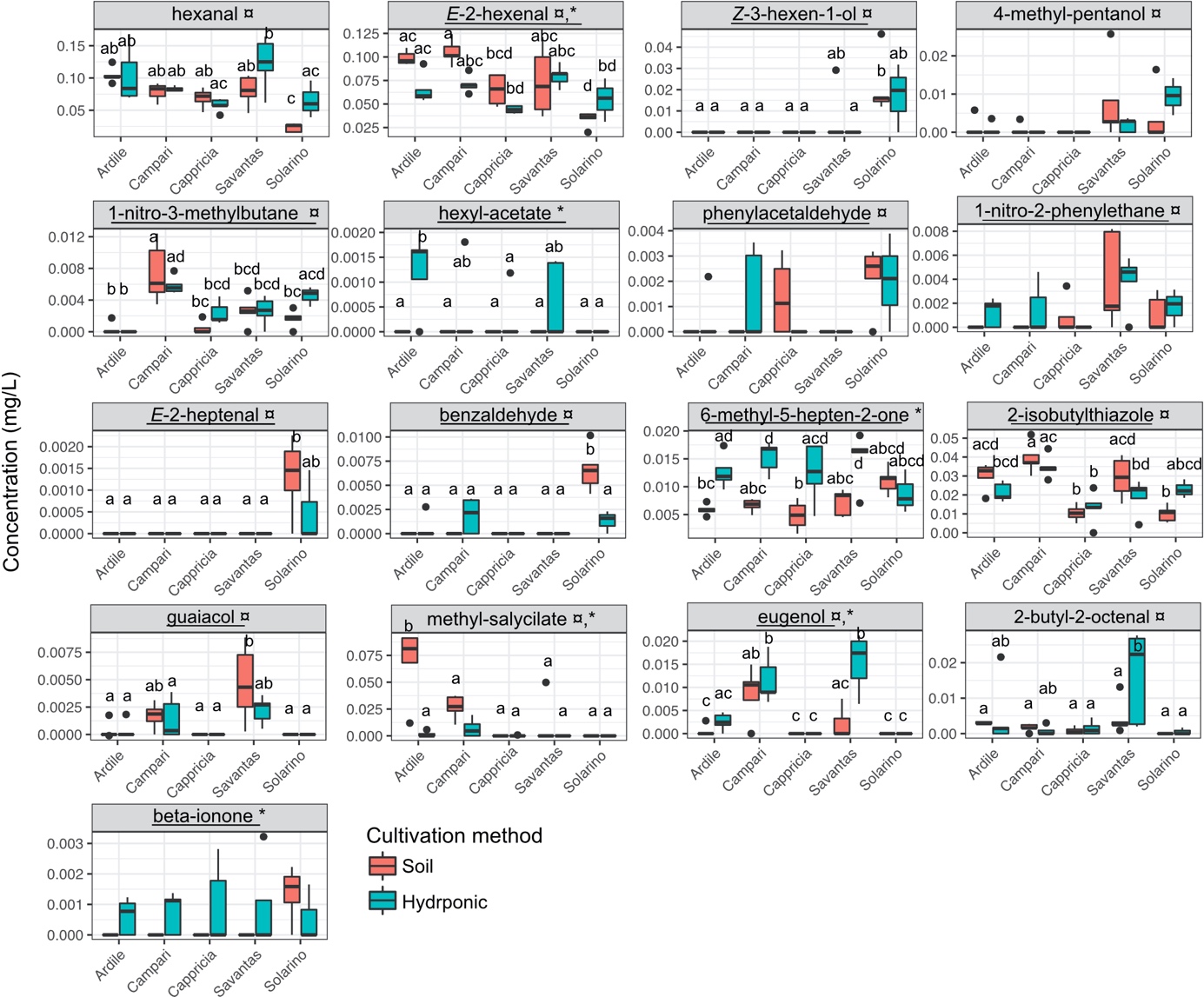

Supplement: Supplementary file 2 [file Table_2.docx]
