# Supplementary material for: The Bacterial Microbiome of the Tomato Fruit Is Highly Dependent on the Cultivation Approach and Correlates With Flavor Chemistry
Source: Front Plant Sci. 2021 Dec 24;12:775722. doi: 10.3389/fpls.2021.775722 (PMC8740158; doi:10.3389/fpls.2021.775722)
Supplement: Supplementary file 3 [file Table_3.docx]

**Supplementary Table 3:** Pairwise comparisons of bacterial community composition among sample types. Ar, Cm, Cp, Sa and Sa stand for ‘Ardiles’, ‘Campari’, ‘Capriccia’, ‘Savantas’ and ‘Solarino’, respectively. Suffixes E and H state whether the samples were cultivated in soil (Einheitserde) or hydroponically, respectively. Underlined P-values show no significant comparisons.

|  | ArE | ArH | CmE | CmH | CpE | CpH | SaE | SaH | SoE |
| --- | --- | --- | --- | --- | --- | --- | --- | --- | --- |
| ArH | 0.012 | - | - | - | - | - | - | - | - |
| CmE | 0.047 | 0.012 | - | - | - | - | - | - | - |
| CmH | 0.012 | 0.012 | 0.012 | - | - | - | - | - | - |
| CpE | 0.012 | 0.012 | 0.012 | 0.012 | - | - | - | - | - |
| CpH | 0.012 | 0.012 | 0.030 | 0.012 | 0.012 | - | - | - | - |
| SaE | 0.012 | 0.012 | 0.012 | 0.012 | 0.012 | 0.012 | - | - | - |
| SaH | 0.012 | 0.012 | 0.012 | 0.019 | 0.012 | 0.209 | 0.012 | - | - |
| SoE | 0.012 | 0.012 | 0.012 | 0.012 | 0.012 | 0.012 | 0.012 | 0.012 |  |
| SoH | 0.012 | 0.065 | 0.012 | 0.061 | 0.012 | 0.153 | 0.012 | 0.363 | 0.012 |
