# Supplementary material for: The Bacterial Microbiome of the Tomato Fruit Is Highly Dependent on the Cultivation Approach and Correlates With Flavor Chemistry
Source: Front Plant Sci. 2021 Dec 24;12:775722. doi: 10.3389/fpls.2021.775722 (PMC8740158; doi:10.3389/fpls.2021.775722)

**Supplementary Figure 2**: Relative abundances of the top 10 bacterial taxa across different taxonomic ranks.


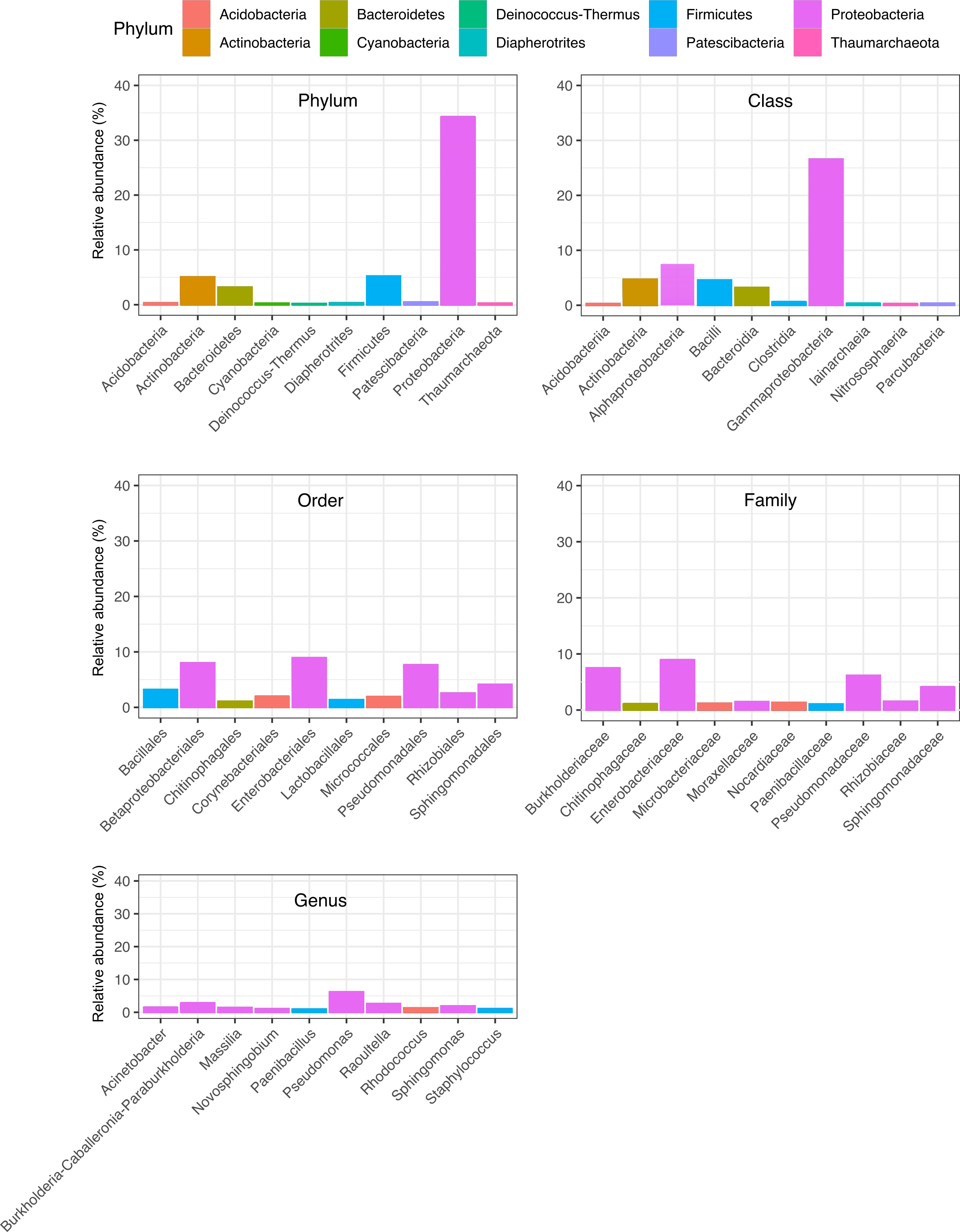

Supplement: Supplementary file 4 [file Table_4.docx]
