# Supplementary material for: The Bacterial Microbiome of the Tomato Fruit Is Highly Dependent on the Cultivation Approach and Correlates With Flavor Chemistry
Source: Front Plant Sci. 2021 Dec 24;12:775722. doi: 10.3389/fpls.2021.775722 (PMC8740158; doi:10.3389/fpls.2021.775722)

**Supplementary Figure 3**: **(A)** Relative abundances of indicator genera per sample type: Ar, Cm, Cp, Sa and Sa stand for ‘Ardiles’, ‘Campari’, ‘Capriccia’, ‘Savantas’ and ‘Solarino’, respectively. Suffixes E and H state whether the samples were cultivated in soil (Einheitserde) or hydroponically, respectively. A total of 46 rASVs classified in 23 genera were identified as indicator species in at least one sample type (Variety:cultivation method combination). **(B)** Occurrence and abundances (log 10) of indicator species per sample type.


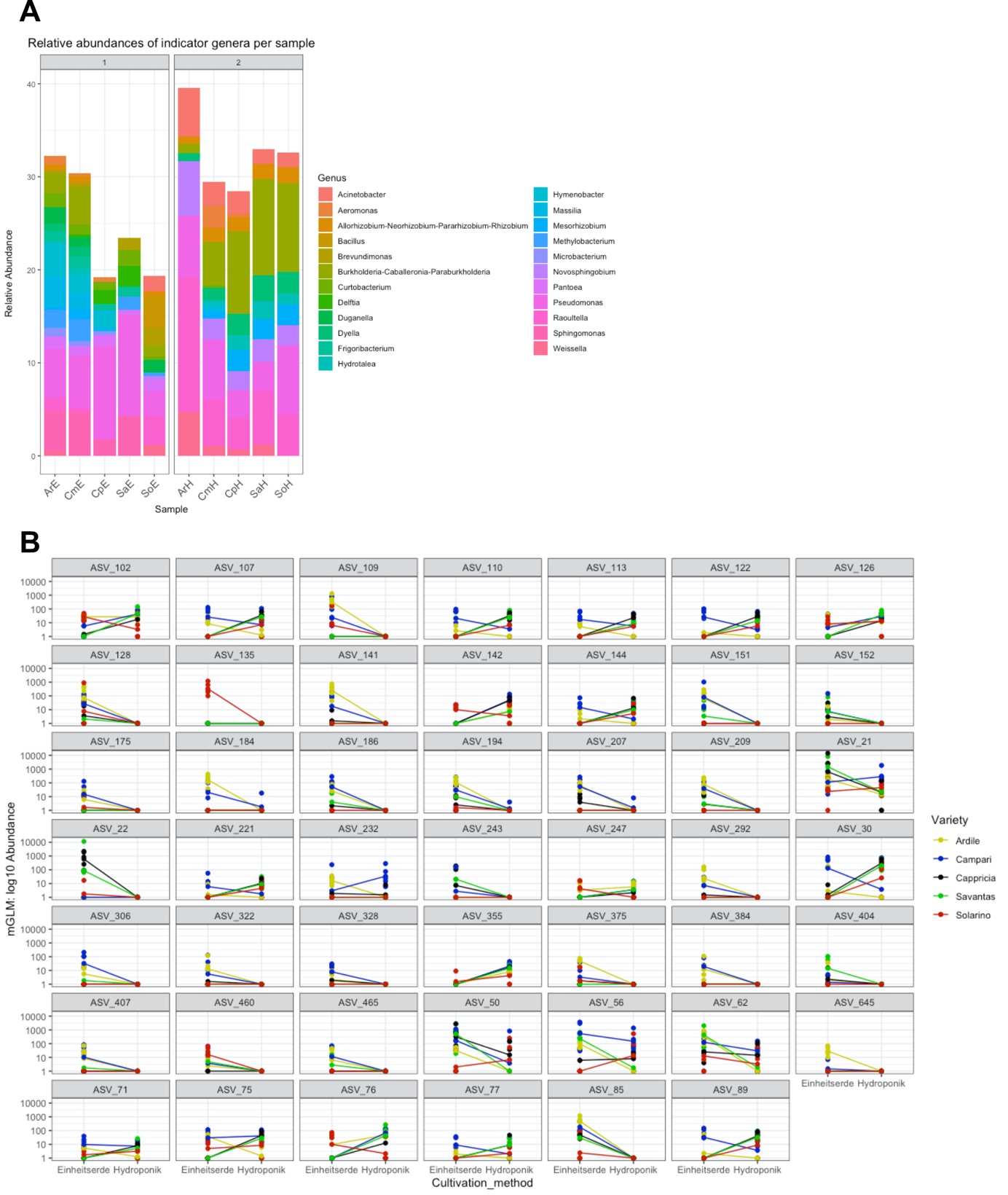

Supplement: Supplementary file 5 [file Table_5.docx]
