# Supplementary material for: The Bacterial Microbiome of the Tomato Fruit Is Highly Dependent on the Cultivation Approach and Correlates With Flavor Chemistry
Source: Front Plant Sci. 2021 Dec 24;12:775722. doi: 10.3389/fpls.2021.775722 (PMC8740158; doi:10.3389/fpls.2021.775722)
Supplement: Supplementary file 7 [file Data_Sheet_2.docx]

**Recipe for Complete Nutrient Solution for young plants (CNS)**

**(pH = 5.6, electric conductivity = 2.5 mS/cm)**

For a 200x Stock solution, amounts in g/L unless indicated otherwise

Calcium nitrate Ca(NO_3_)_2_ ……………………………………192.5

Potassium nitrate KNO_3_……………………………………….2.5

Ammonium Nitrate NH_4_NO_3_………………………………….10 ml

Iron chelate EDDHMA 6%.......................................................6.5

Phosphoric acid 75%..................................................................25 ml

Potassium nitrate KNO_3_……………………………………… 57.5

Ammonium dihydrogen phosphate (NH_4_)H_2_PO_4_……................ 5

Potassium sulfate K_2_SO_4_……………………………………….47.5

Magnesium sulfate MgSO_4_…………………………………….102.5

Manganese sulfate MnSO_4_..........................................................0.4

Zink sulfate ZnSO_4_......................................................................0.275

Sodiumtetraborat (Borax)...........................................................0.7

Copper sulfate CuSO_4_………………………………………….0.05

Sodium molybdate NaMoO_4_………………………………….0.025

**Recipe for Complete Nutrient Solution for cocopeat mats (CNS)**

**(pH = 5.6, electric conductivity = 3.5 mS/cm)**

Calcium nitrate Ca(NO_3_)_2_ ……………………………………265

Ammonium Nitrate NH_4_NO_3_………………………………….5 ml

Iron chelate EDDHMA 6%.......................................................6.5

Phosphoric acid 75%..................................................................25 ml

Potassium nitrate KNO_3_……………………………………… 80

Ammonium dihydrogen phosphate (NH_4_)H_2_PO_4_……................ 5

Potassium sulfate K_2_SO_4_……………………………………….30

Magnesium sulfate MgSO_4_…………………………………….152.5

Manganese sulfate MnSO_4_..........................................................0.5

Zink sulfate ZnSO_4_......................................................................0.325

Sodiumtetraborat (Borax)...........................................................1.275

Copper sulfate CuSO_4_………………………………………….0.05

Sodium molybdate NaMoO_4_…………………………………...0.05
